# A Study on the Motion Behavior of Metallic Contaminant Particles in Transformer Insulation Oil under Multiphysical Fields

**DOI:** 10.3390/s24175483

**Published:** 2024-08-24

**Authors:** Binbin Wei, Zhijuan Wang, Runze Qi, Xiaolong Wang, Tong Zhao

**Affiliations:** School of Electrical Engineering, Shangdong University, Jinan 250061, China; weibinb@mail.sdu.edu.cn (B.W.); wangzhijuan@sdu.edu.cn (Z.W.); qirz16@mail.sdu.edu.cn (R.Q.); wangxiaolong@sdu.edu.cn (X.W.)

**Keywords:** transformer, flowing insulating oil, different electric fields, metallic impurity particle, motion characteristic simulation

## Abstract

When using transformer insulation oil as a liquid dielectric, the oil is easily polluted by the solid particles generated in the operation of the transformer, and these metallic impurity particles have a significant impact on the insulation performance inside the power transformer. The force of the metal particles suspended in the flow insulation oil is multidimensional, which will lead to a change in the movement characteristics of the metal particles. Based on this, this study explored the motion rules of suspended metallic impurity particles in mobile insulating oil in different electric field environments and the influencing factors. A multiphysical field model of the solid–liquid two-phase flow of single-particle metallic impurity particles in mobile insulating oil was constructed using the dynamic analysis method, and the particles’ motion characteristics in the oil in different electric field environments were simulated. The motion characteristics of metallic impurity particles under conditions of different particle sizes, oil flow velocities, and insulation oil qualities and influencing factors were analyzed to provide theoretical support for the detection of impurity particles in transformer insulation oil and enable accurate estimations of the location of equipment faults. Our results show that there are obvious differences in the trajectory of metallic impurity particles under different electric field distributions. The particles will move towards the region of high field intensity under an electric field, and the metallic impurity particles will not collide with the electrode under an AC field. When the electric field intensity and particle size increase, the trajectory of the metallic impurity particles between electrodes becomes denser, and the number of collisions between particles and electrodes and the motion speed both increase. Under the condition of a higher oil flow velocity, the number of collisions between metal particles and electrodes is reduced, which reduces the possibility of particle agglomeration. When the temperature of the insulation oil changes and the quality deteriorates, its dynamic viscosity changes. With a decrease in the dynamic viscosity of the insulation oil, the movement of the metallic impurity particles between the electrodes becomes denser, the collision times between the particles and electrodes increase, and the maximum motion speed of the particles increases.

## 1. Introduction

The safe and reliable operation of large power transformers, the cornerstone of the UHV power grid, is crucial to ensuring the overall safety of the entire power grid [1]. At present, large power transformers are generally oil-immersed structures, and their interior is generally combined oil–paper insulation. The insulating medium includes insulating oil, paper, cardboard, and other solid insulating components. Insulating oil serves as both an insulator and a coolant, and its quality significantly affects the insulation performance of the oil paper. In practical engineering applications, insulation oil often contains various types of impurities due to issues related to manufacturing, transportation, on-site filling, operation, and maintenance. These impurities can include metal particles, air bubbles, moisture, fibers, paper fragments, and other contaminants [2,3]. In the strong-gradient electric field environment, these impurity particles in the insulation oil will accumulate and may form a small bridge, which will induce discharge and deteriorate the insulation performance of the transformer [4,5]. Among them, the proportion of fiber impurity particles is the largest. Therefore, the influence of fiber impurity particles on the insulation properties of insulation oil has been studied extensively by domestic and international scholars. Reference [6] studies the effects of voltage amplitude, caking degree, and oil flow velocity on the caking and partial discharge characteristics of fiber particles from an experimental point of view. In real-world engineering applications, insulation oil frequently harbors a range of impurities resulting from manufacturing, transportation, on-site filling, operations, and maintenance processes. These impurities may comprise metal particles, air bubbles, moisture, fibers, paper debris, and other contaminants [7,8]. Therefore, timely monitoring and tracking of changes in these particle impurities in the insulation oil helps discover the hidden risks in the transformer and accurately determine the location of the fault in a timely manner.

Extensive research on metal particles in insulation oil has been conducted both domestically and internationally, with a primary focus on how these particles impact the breakdown voltage of the insulation oil [9,10]. The authors of [11,12] investigated the effects of conductive and non-conductive particle impurities on the breakdown characteristics of insulating oil under AC voltage. Their findings revealed that conductive particles have a more significant impact on the breakdown voltage of the insulating oil. The authors of [13] employed simulations to examine the influence of conductive particles on the insulation properties of insulating oil under AC voltage. The analysis concluded that conductive particles play a crucial role in the breakdown process of insulating oil that is contaminated with impurities. The authors of [14] investigated the influence of metal particles on the power frequency breakdown voltage of insulating oil. The study found that, as the median particle size of metal particles introduced into the insulating oil increases, the corresponding breakdown voltage decreases. Research on the motion characteristics of metal particles in insulating media primarily focuses on the behavior of particles in stationary insulating oil. S. Barlasekaran from Australia studied the movement of metal spheres between vertical flat electrodes under a direct current electric field [15]; researchers from the University of Genoa in Italy examined the movement of metal particles between ball and plate electrodes in both DC and AC fields [16,17]; C. Cohoi et al. investigated the motion of metal particles between plate electrodes under both uniform and non-uniform electric fields [18]; and building on this foundation, Chinese scholars, including Wang Youyuan from Chongqing University, studied the motion rules of metal particles between plate electrodes, pin–plate electrodes, and double-ball electrodes under different ratios of AC/DC combined voltage [19]. For stationary insulating oil, the electric field predominantly governs the movement of metal particles [20]. This includes the electric field force on charged metal particles and the dielectrophoretic force effect in a non-uniform electric field [21]. However, in large power transformers, the internal insulating oil generally utilizes forced-oil-circulation air cooling or forced-oil-circulation-directed air cooling, leading to a continuous flow state with typical oil flow velocities below 1 m/s [22]. Under these conditions, the force acting on the metallic impurity particles suspended in flowing insulating oil is multidimensional, which alters their motion characteristics. Currently, there is limited research on the impact of flowing insulating oil on the motion characteristics of metallic impurity particles.

In response to this, this paper investigates the motion dynamics of and influencing factors on suspended metallic impurity particles in flowing insulating oil under various electric field conditions. Utilizing dynamic analysis methods, a multiphysical field model was developed to represent the solid–liquid two-phase flow of single metallic impurity particles in flowing insulating oil. Simulations were conducted to explore the motion characteristics of these particles across different electric field environments. This study analyzed how variables such as particle size, oil flow velocity, and insulation oil quality affect particle behavior. The findings offer theoretical insights into the detection of impurity particles in transformer insulation oil and the precise identification of the location of equipment faults.

## 2. Kinetic Simulation Analysis of Metallic Impurity Particles in Flowing Insulation Oil

### 2.1. Kinetic Analysis of Metallic Impurity Particles

Insulating oil usually circulates inside power equipment, and the movement of particulate matter in the impurity phase is affected by the combined factors of flow, electricity, and heat field, while the most important factor determining its movement is the force of the particulate matter. The forces acting on metallic impurity particles in insulating oil arise from several sources. The electric field exerts both an electric field force, due to the charge on the impurity particles, and an electrophoretic force, resulting from particle polarization. The thermal field contributes a thermo-swimming force, which arises from the effect of the temperature gradients on particle collisions. In the flow field, the primary force is the drag force exerted by the insulating oil on the impurity particles, which is closely linked to the fundamental properties of the two-phase flow. Additionally, particles experience other forces such as gravity, buoyancy, added mass inertia, the Basset force, the Magnus lift, the Saffman force, and the pressure gradient force. These additional forces are typically influenced by the particle’s sphericity and spin velocity during motion.

The main forces of particulate matter in the impurity phase in insulation oil are shown in Table 1 below [23].

Each parameter in Table 1 is limited by the characteristic structure of the simplified particle motion model in the plate electrode. In practice, if the research object is complex and the model is inconsistent, the model’s difference may affect the expression form and even the relative magnitude of the main force. As illustrated in Table 1, it is generally accepted that, when the particle size of impurity phase particles is within the micrometer range, the drag force and electric field force are the predominant factors. In contrast, the Magnus force primarily serves to counterbalance the effect of gravity. As the particle size decreases and fluid flow becomes more stable, the relative significance of the additional mass inertia force, Basset force, and pressure gradient force diminishes significantly. Similarly, the influence of the Saffman and buoyancy forces also decreases. When there are substantial differences in magnitude, certain forces can be disregarded to simplify the analysis. Besides the drag and electric field force, it is also important to account for the electrophoretic force in non-uniform electric fields, as this force arises due to the significant electric field gradient near regions of high field intensities.

Therefore, in this study, the electric field force (*F_e_*), gravity (*G*), buoyancy (*F*), oil viscous resistance (*F*_0_), dielectrophoretic force (*F_dep_*), drag force (*F_D_*), and other forces were mainly considered when establishing the dynamic trajectory simulation model of metallic impurity particles in flowing insulating oil, in which gravity and buoyancy act as opposing forces.

As charged metal particles move towards the electrode, they are primarily influenced by the electric field force *F_e_* [24], whose expression is as follows:

(1) $$F_{e}=qE$$

(2) $$q=\frac{2}{3}\pi^{3}\varepsilon_{m}\varepsilon_{0}r^{2}E$$

where *E* is the electric field intensity of the environment in which the metallic impurity particles are located; *q* is the charge carried by the metallic impurity itself. *q* is related to the particle size, the relative permittivity of the liquid medium, and the applied electric field’s strength. $\varepsilon_{m}$ and $\varepsilon_{0}$ are the relative permittivity of the liquid medium and the vacuum permittivity.

When the type and size of metallic impurity particles and the type of liquid medium are determined, the magnitudes of the gravitational and buoyant forces on the particles are also determined. Therefore, when examining these forces on metal impurities, the resultant forces of gravity and buoyancy are considered together, denoted as *F_m_*, and the formula is as follows:

(3) $$F_{m}=m_{0}g\frac{\rho_{1}-\rho_{2}}{\rho_{1}}$$

where $\rho_{1}$ is the density of the insulating oil; $\rho_{2}$ is the density of the metallic impurity particles; $m_{0}$ represents the mass of the metallic impurity particles; and g denotes the acceleration due to gravity.

The force exerted by the fluid on an object in the case of relative motion between the object and the fluid is called the fluid drag force [25]. The drag force exerted by the oil flow on the metallic impurity particles, which acts in the direction of the relative motion of the metallic impurity particles, can be calculated using the following formula:

(4) $$\left\{ \begin{array}{l} F_{D}=\frac{1}{\tau_{p}}m_{0}(u-v)\\ \tau_{p}=\frac{\rho_{p}{d_{p}}^{2}}{18\eta_{oil}} \end{array}\right.$$

where *u* is the fluid flow velocity; $\rho_{p}$ is the density of the metallic impurity particles; $d_{p}$ is the diameter of the metallic impurity particles; and $\eta_{oil}$ is the fluid viscosity.

Transformer insulating oil generally possesses a certain viscosity. When metallic impurity particles that are immersed in it move, their surfaces will adhere to some viscous oil. This oil will follow the particles as they move, resulting in a certain internal friction force between the two. This force is called the oil viscous resistance [21]. According to the classic Stokes model of the viscous resistance experienced by particles in a liquid, the viscous resistance *F_0_* experienced by metallic impurity particles in insulating oil can be calculated as follows:

(5) $$F_{0}=6\pi \eta_{oil}rv$$

where $\eta_{oil}$ is the viscosity of the oil; v is the velocity of the particle.

Dielectrophoresis is mainly used to describe the directional movement of particles suspended in a liquid under the action of a non-uniform electric field due to electric field polarization [26]. Any particle that is suspended in an electric field will become polarized under the action of an electric field and produce a free charge in the object. The *F_dep_* formula for the dielectrophoresis force on the particles in the oil is as follows:

(6) $$F_{dep}=2\pi r^{3}\varepsilon_{m}(\frac{\sigma_{p}-\sigma_{oil}}{\sigma_{p}+2\sigma_{oil}})\nabla E^{2}$$

where $\varepsilon_{m}$ is the relative dielectric constant of the liquid medium, and $\sigma_{p}$ is the conductivity of the particle, while $\sigma_{oil}$ is the conductivity of the oil medium.

In summary, the kinetic equation for metallic impurity particles in flowing insulating oil can be obtained as follows:

(7) $$ma=F_{e}+F_{m}+F_{D}+F_{0}+F_{dep}$$

It can be seen from the kinetic equation for the metallic impurity particles that their motion state will be affected by the distribution of the fluid field. Similarly, the reaction force of the movement of the metallic impurity particles on the fluid cannot be ignored, so the particles and insulating oil exhibit a solid–liquid coupling state. The insulating oil is applied to the metallic impurity particles by means of the drag force, buoyancy force, and other forces, and this is reflected in the movement track of the metallic impurity particles. The metallic impurity particles react on the insulation oil through the reaction force, that is, the external force in the equation, and are reflected in the distribution of the fluid field.

### 2.2. Analysis of Solid–Liquid Two-Phase Flow

To analyze the dynamic behavior and basic motion characteristics of metallic impurity particles in the oil, the key is to model and solve the liquid–solid two-phase flow problem under the coupled condition of flow, electricity, and heat. Lagrange analysis and the Euler method are commonly used to study fluid motion in the field of fluid mechanics.

According to the basic theory of liquid–solid two-phase flow, when the volume fraction of the solid phase is greater than 10~12%, the liquid–solid two-phase flow of dense particles is usually described in Euler–Euler coordinates using the Euler–Euler model as a reference [27,28]. When the volume fraction of the solid phase is below 10–12%, it belongs to the liquid–solid two-phase flow of sparse particles, and it is necessary to describe the solid phase in Lagrangian coordinates, while the liquid phase should be described in a Euler coordinate system. In this case, it is more appropriate to establish the conservation equations for mass, momentum, and energy, and the flow belongs to the Euler–Lagrangian model [29].

In this study, a fluid flow particle tracking model is employed to analyze the motion of the metallic impurity particles. The Lagrangian method is applied to compute the solid phase, while the Eulerian method is used for the fluid phase. The motion equations for the metallic impurity particles are coupled with the Navier–Stokes equations for the fluid phase to determine the particle velocities. Subsequently, integrating the particle velocities over time provides the trajectory of the particles. This approach offers computational simplicity and effectively handles complex particle trajectories, provided that the number of particles remains manageable, which aligns with the simulation requirements of this study.

## 3. Finite Element Simulation Model of Metal Particle Movement

### 3.1. The Establishment of the Simulation Model

**Figure 1:**
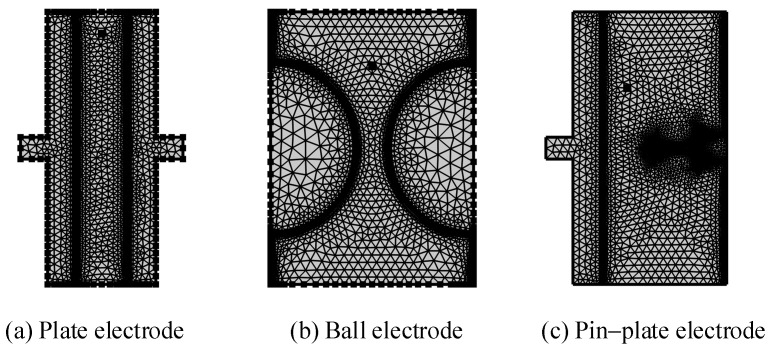
Schematic diagram of 2D simulation segmentation.

The low-voltage winding oil channel spacing in the large transformer ranges from 1.823 mm to 8 mm, the vertical oil channel size is 8 mm, and the horizontal oil channel has three sizes of 1.823 mm, 3.647 mm, and 5.470 mm [30]. In order to simulate the trajectories of the metallic impurity particles under different electric fields, three electrode models were constructed in this paper, which were plate electrode, ball electrode, and pin–plate electrode. When choosing the three electrode models of plate electrode, ball electrode, and pin–plate electrode, the following factors were mainly considered: Different shapes of electrodes will affect the distribution and intensity of the electric field [31], because the geometry of the electrode determines the distribution mode and intensity of the electric field. A plate electrode is usually used to generate a uniform electric field and is suitable for applications where parallel electric fields are required. A ball electrode produces a relatively concentrated and symmetrical electric field and is suitable for applications where a uniform central electric field is required. A pin–plate electrode has a high local electric field strength and is suitable for applications that require a concentrated electric field. The transformer tank environment contains a variety of uniform and non-uniform electric fields. For example, the tap-changer and tank wall in the transformer’s on-load tap-changer will form a variety of non-uniform electric fields [32]. Therefore, these three electric fields can cover various electric field environments in practical applications. Among them, the plate electrode uses a copper plate electrode with a diameter of 25 mm and a thickness of 2.5 mm. The ball electrode is composed of a ball electrode with a diameter of 25 mm. The plate electrode part of the pin–plate electrode is consistent in size, the diameter of the needle electrode part is 1 mm, and the needle tip’s amplitude is 200 μm. In order to fully consider the movement of the metallic impurity particles between the electrodes, the spacing of the simulated electrodes was set at 5 mm. At the same time, due to the symmetrical structure of the electrode, a 2D model was used to construct the electrode in order to reduce the workload during the calculation process. The configuration of the 2D model following segmentation is depicted in Figure 1, and the material properties utilized in the model are detailed in Table 2.

In the simulation process, the main purpose is to analyze the force model of the external forces on the metallic impurity particles under the electric field. It is assumed that the collision of the metallic impurity particles on the electrode plate is elastic, and the time when the metallic impurity particles’ contact with the plate is full of charge is ignored.

### 3.2. Adding a Physical Field

#### 3.2.1. Current Field

Based on Maxwell’s equations, the finite element equation in the electric field is expressed as follows:

(8) $$\nabla \cdot J=Q_{j}$$

(9) $$J=\sigma E+j\omega D+J_{e}$$

(10) E=−∇V

where *J* is the current density; $Q_{j}$ is the changed amount of charge; *E* is the electric field intensity; σ is the conductivity; σE is the conduction current; ω is the angular frequency; *D* is the electric displacement vector, which means that jωD is the displacement current; $J_{e}$ is the applied current density; and *V* is the potential.

In Formula (8), the term represents the divergence of the electric current, which corresponds to the rate of change in charge density. Formula (9) indicates that the total current consists of the conduction current, the displacement current, and any applied current. Additionally, in Formula (10), the electric field strength is defined as the negative gradient of the electric potential.

#### 3.2.2. Fluid Field

The fluid in the oil passage of the transformer studied in this paper exhibits a low velocity and simple motion, which is more appropriately described using the standard k-ε turbulence model. The theoretical study of fluid mechanics shows that any fluid flow and heat transfer follow the three conservation laws of mass, momentum, and energy. If it is a turbulent flow, the Reynolds average of each physical quantity in the flow field is processed in the time domain, and the time-homogenized Reynolds average compressible N-S equations are obtained [33].

(11) $$\left\{ \begin{array}{l} \frac{\partial \bar{\rho }}{\partial t}+\frac{\partial \bar{\rho }{\bar{u}}_{i}}{\partial x_{i}}=0\\ \frac{\partial (\bar{\rho }{\bar{u}}_{i})}{\partial t}+\frac{\partial (\bar{\rho }{\bar{u}}_{i}{\bar{u}}_{j})}{\partial x_{j}}=-\frac{\partial \bar{\rho }}{\partial x_{i}}+\frac{\partial \bar{\rho }}{\partial x_{j}}({\bar{\tau }}_{ij}-\rho {{\bar{u}}_{i}}^{\prime \prime }{u_{j}}^{\prime \prime })\\ \frac{\partial (\bar{\rho }E)}{\partial t}+\frac{\partial (\bar{\rho }{\bar{u}}_{j}H)}{\partial x_{j}}=-\frac{\partial }{\partial x_{j}}(-q_{Lj}-q_{Tj}+\tau_{ij}{{\bar{u}}_{i}}^{\prime \prime }-\frac{1}{2}\rho {u_{j}}^{\prime \prime }{{\bar{u}}_{i}}^{\prime \prime }{u_{i}}^{\prime \prime })+\frac{\partial }{\partial x_{j}}({\bar{u}}_{i}({\bar{\tau }}_{ij}-\rho {{\bar{u}}_{i}}^{\prime \prime }{u_{j}}^{\prime \prime })) \end{array}\right.$$

Here, ρ denotes the fluid density; *p* represents the pressure; *E* is the internal energy; *H* is a dimensionless parameter; $u_{i}$ and $u_{j}$ are the Reynolds-averaged velocity components; ${u_{i}}^{\prime \prime }$ and ${u_{j}}^{\prime \prime }$ are the fluctuating velocity components; $\tau_{ij}$ is the stress tensor component; $q_{Lj}$ is the laminar heat flow; and $q_{Tj}$ is the turbulent heat flow.

#### 3.2.3. Fluid Flow Particle Tracking

The process of external forces impacting the movement of metallic impurity particles in a liquid medium is consistent over time, and the movement of particles at each moment is obviously affected by the movement of the previous moment. The fluid flow particle tracking module can track the movement of a certain particle in real time, calculate the force of the particle and the change law of the movement speed at each time point, and calculate the resulting movement of the particle cumulatively in time. In this paper, based on the force analysis results for the suspended metallic impurity particles in insulation oil, the gravity, buoyancy, viscous resistance, electric field force, drag force, and dielectrophoretic force are taken as the main forces on the metallic impurity particles in order to carry out a force analysis on metallic impurity particles, and then, the relationship between the moving speed of the metallic impurity particles and the resultant force is obtained. The motion model of the metallic impurity particles can then be derived, as shown in Equation (12).

(12) $$\frac{d}{dt}(m_{p}v)=F_{e}+F_{m}+F_{0}+F_{D}+F_{dep}$$

Here, $F_{e}$, $F_{m}$, $F_{0}$, $F_{D}$, and $F_{dep}$, respectively, represent the electric field force, the combined force of gravity and buoyancy, viscous resistance, drag force, and the electrophoretic force of the metallic impurity particles.

## 4. Analysis of Movement Characteristics of Metallic Impurity Particles in Flowing Insulating Oil

In this paper, the motion characteristics of particles in insulating oil are studied by comparing and analyzing the particle motion trajectory, particle velocity, and number of collisions between particles and the plate. On the one hand, the metallic impurity particles are suspended in the insulating oil and are in contact with the surface of the electrode, meaning that the metallic impurity particles themselves will carry a charge of the same polarity as the electrode due to the transfer of charge. When the particle is charged, it will be subject to the repulsive force of the local electrode and the attractive force of the negative electrode, and under the combined action of the two forces, it will move towards the opposite-polarity electrode. When the particles arrive at the opposite electrode, the charge on the particles will neutralize and take on a heteropolar charge, and a charge neutralization and charging process will occur on the particles, which is manifested as a short pulse current between the particles and the electrode [24]. When the particle is full of charge, it will move toward the previous electrode. When there is no external interference between the electrodes, the particle will repeat this collision process with the electrode without restriction, meaning that the particle will transfer currents between the electrodes as a carrier of charge. Frequent particle collisions with electrodes may greatly increase the probability of discharge, resulting in insulation damage [34]. On the other hand, the motion speed of the particles affects their kinetic energy, and high-speed particles will have a greater impact during the collision, which may lead to a local arc or thermal effect, thereby reducing the insulation strength of the insulating oil. The change in particle velocity also draws on the change in the number of collisions between the particle and plate. The particle trajectories affect the electric field distribution in the insulating oil. Certain trajectories can lead to a concentration of electric fields, increasing the local field strength and thus increasing the risk of breakdown. Therefore, it is necessary to analyze the particle trajectory, particle velocity, and number of collisions between the particle and the plate, as these are important characteristics.

### 4.1. Effect of Electric Field Distribution on Motion of Metallic Impurity Particles

**Figure 2:**
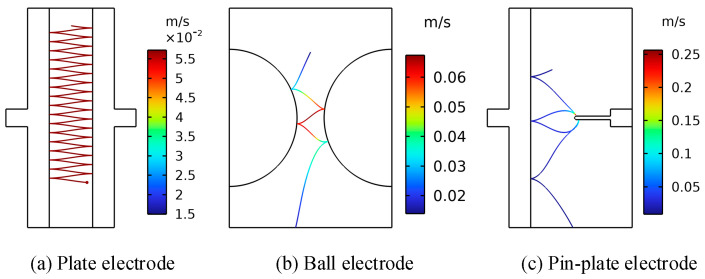
Motion trajectories of metallic impurity particles under different electrodes.

**Figure 3:**
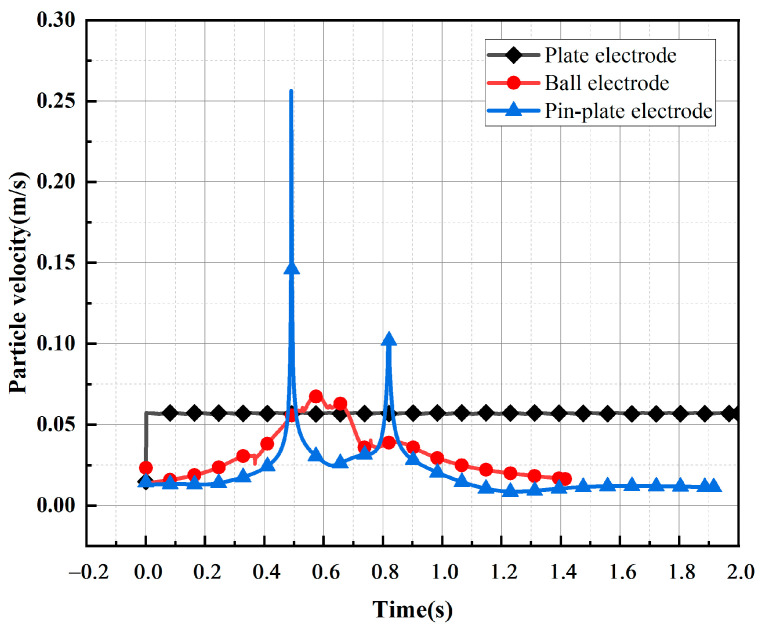
A comparison of the velocities of the metallic impurity particles under different electrodes.

In order to verify the influence of the electric field distribution environment on the motion of metallic impurity particles, the trajectories of these particles under three electrode structures were simulated. In the simulation process, the electrode voltage was set as a DC voltage of 10 kV, and the diameter of the metallic impurity particles was 50 μm. Because the oil flow velocity distribution in the transformer was very uneven and parabolic, the highest speed was 1.5 m/s, while the lowest speed was 0.01 m/s [35]. Here, we set the insulation oil flow rate to a constant value of 0.01 m/s. The velocity variation of the metallic impurity particles over 3.0 s was calculated using a simulation. Figure 2 shows the trajectory diagram of the metallic impurity particles under different electrodes. Figure 3 shows the velocity changes of the metallic impurity particles under different electrodes.

As illustrated in Figure 2, the metallic impurity particles migrate towards the regions of higher electric field strengths between the electrodes due to the influence of the electric field. Additionally, the varying electric field distribution among the three electrodes leads to markedly different trajectories of the solid particles between them. Combining Figure 3 and Table 3, it can be seen that the motion process of the particles under the plate electrode is relatively stable and regular. Throughout the process, the maximum particle motion velocity is 0.0573 m/s, while the average particle motion velocity remains consistent at 0.0570 m/s. Meanwhile, the overall velocity of the particles fluctuates a little. When the particles are close to the surface of the ball electrode, the motion velocity is higher, and the maximum motion velocity reaches 0.0674 m/s. When the particles are far away from the surface of the ball electrode, the motion velocity decreases to a certain extent. The average velocity of the particles during the whole motion process is 0.0320 m/s, and the velocity fluctuation amplitude is large. It can be concluded that the motion velocity of the particles between spherical electrodes is caused by the distribution of electric fields between the spherical electrodes, which is low on both sides and high in the middle. Under the pin–plate electrode, the motion velocity of the metallic impurity particles changes the most. The maximum velocity of particle motion is 0.2565 m/s, and the average velocity is only 0.02 m/s. This is because the high-electric-field area under the pin–plate electrode is mainly concentrated near the tip of the needle, and particles will move towards the tip of the needle after colliding with the plate electrode.

### 4.2. Effect of Electric Field Intensity on Motion of Metallic Impurity Particles

In order to analyze the influence of the electric field strength on the motion of solid particles, the trajectories of metallic impurity particles under 5 kV, 12 kV, and 15 kV voltages were calculated and compared with those under 10 kV. In the simulation process, the diameter of the metallic impurity particles was set to 50 μm, and the oil flow velocity was set to 0.01 m/s. Plate and ball electrodes were selected during the simulation.

**Figure 4:**
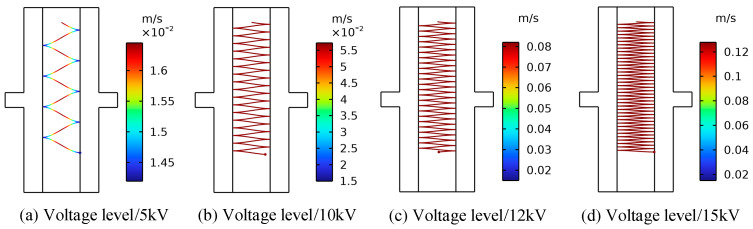
Motion trajectories of metallic impurity particles in the plate electrode under different electric field intensities.

**Figure 5:**
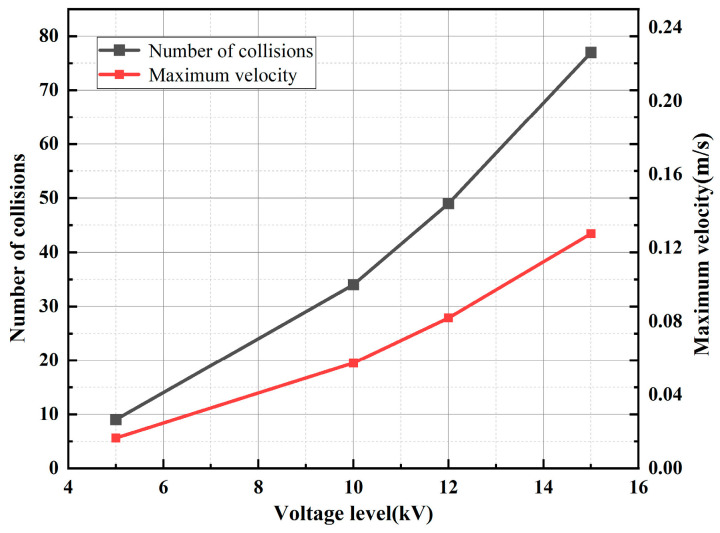
Characteristic motion parameters of metallic impurity particles in plate electrode under different electric field intensities.

Figure 4 and Figure 5, respectively, show the trajectory diagram of the metallic impurity particles in the plate electrode and the comparative diagram of the characteristic motion parameters of the metallic impurity particles using the plate electrode when different voltages are applied. It can be seen from the figures that with the increase in voltage level, the particles’ movement trajectory between electrodes becomes increasingly dense. The maximum particle velocity and frequency of collisions with the plate increase in direct proportion to the applied voltage. At the same time, the maximum velocity of the metal particles between electrodes and the number of collisions between particles and electrodes increase significantly.

**Figure 6:**
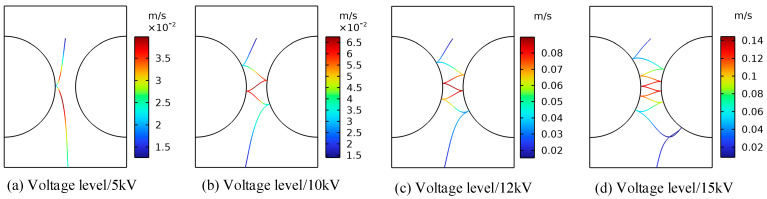
Motion trajectories of metallic impurity particles under different electric field intensities in spherical electrode.

**Figure 7:**
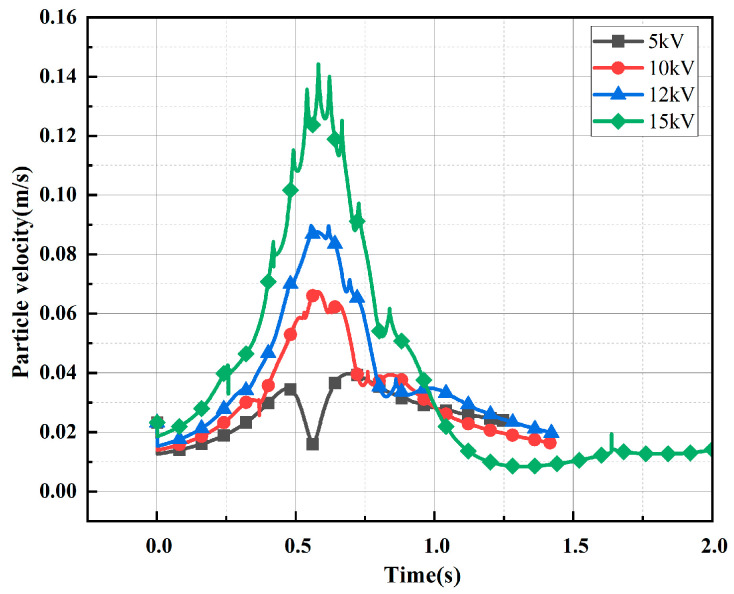
Comparison of metal particle velocities under different electric field intensities.

Figure 6 shows the trajectory diagram of metallic impurity particles under different electric field strengths in the ball electrode. The trajectory of the metallic impurity particles in the ball electrode is highly similar to that of the plate electrode under different electric field intensities. With the increase in the external electric field intensity, the trajectory of the metallic impurity particles between the ball electrodes becomes increasingly dense, but the particles move out of the model boundary under the ball electrode in advance. It can be seen from Figure 7 and Table 4 that, under different voltage levels, the particle velocity reaches its maximum at the position of the central axis of the ball electrode and then decreases. When a 5 kV voltage is applied, the maximum velocity of the particles is 0.0398 m/s, and the average velocity is 0.0266 m/s. When a voltage of 15 kV is applied, the maximum particle motion velocity increases to 0.1443 m/s, and the average velocity is 0.0352 m/s, which is much higher than the motion velocity of the particles when a low voltage is applied. At the same time, as the particle velocity increases, the longitudinal advance time of the particle becomes longer. When a 5 kV voltage is applied, the particles have already moved to the boundary of the model at 1.245 s. With the increase in the applied voltage level, the time for the particles to move to the boundary of the model becomes increasingly long. At 15 kV, the time required reaches 2.387 s.

Based on the analysis of the results, this can be attributed to the fact that the metallic impurity particles are predominantly influenced by the electric field force between the plates. According to Equations (1) and (2), the magnitude of the electric field force on the metallic impurity particles is proportional to the square of the electric field strength. As the electric field strength increases, the electric field force on the particles also increases, leading to an acceleration in the transverse motion speed of the particles. Consequently, the trajectories of the particles between the plates become increasingly dense, which in turn results in a higher frequency of collisions between the impurity particles and the electrode. As a result, it takes longer for the particles to reach the axial boundary.

### 4.3. The Effect of Voltage Type on the Movement of Metallic Impurity Particles

In contrast to an ordinary AC transformer, the main insulation of the converter transformer also withstands DC and AC/DC combined voltage [36,37]. The motion characteristics of the particles in the oil are significantly different from those under ordinary AC, resulting in a significant difference in the breakdown voltage of the insulating oil. Therefore, to study the motion distribution characteristics of the metal particles under AC and DC voltages and the impact on the breakdown strength of the insulating oil, as well as the impact of the control particles on the insulation performance of the converter transformer, it is of great significance to improve the safe and stable operation of converter transformers [38]. In order to analyze the influence of the voltage type on the movement of metallic impurity particles, the trajectories of metallic impurity particles under AC voltage, DC voltage, and AC/DC combined voltage (1:2) were simulated. In the simulation process, the diameter of the metallic impurity particles was set as 50 μm, and the oil flow velocity was set as 0.01 m/s. Plate and ball electrodes were selected for the simulation. Due to the large amount of calculation required for the simulation under AC voltage, the simulation time in this section was shortened to 1 s to shorten the calculation time.

**Figure 8:**
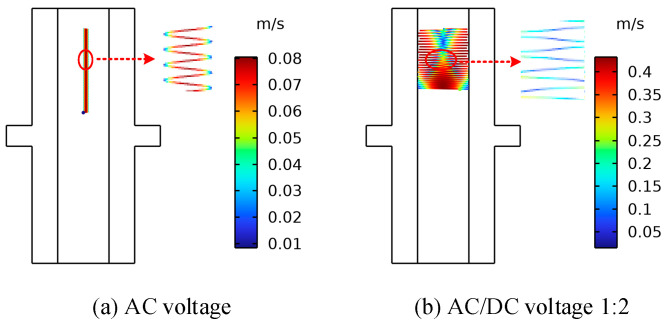
The trajectory diagrams of metallic impurity particles under different voltage types in the plate electrode.

**Figure 9:**
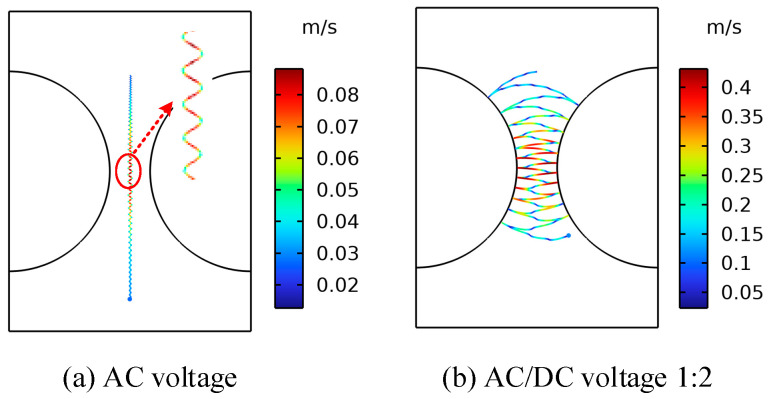
Trajectories of metallic impurity particles under different voltage types in ball electrode.

Figure 8 and Figure 9 show the motion paths of the metallic impurity particles under different voltage types in the plate electrode and the ball electrode, respectively. The motion paths of the metallic impurity particles under the two types of electrodes are essentially the same. There are obvious differences in the motion trajectories of solid particles under AC voltage, DC voltage, and AC/DC combined voltage. Under AC voltage, the solid particles do not collide with electrodes. However, under DC voltage and AC/DC combined voltage, the solid particles follow the movement law of collision between positive and negative electrodes. At the same time, it can be seen from the local magnification of the trajectory in Figure 8a that the motion trajectory of the particles in the AC electric field environment swings from side to side within a period of 20 ms, and the overall motion trajectory is a straight line in the direction of gravity. Our analysis shows that the motion velocity of metallic impurity particles under the action of an electric field is proportional to the force of the electric field. Under AC voltage, the electric field strength between the electrodes changes according to the sine function, so the electric field force that is subjected to the particles also changes periodically. Finally, the solid particles do not show a trend of directional movement towards the electrodes under AC voltage, but they do periodically swing between electrodes.

According to the data in Table 5, the maximum motion velocity, average motion velocity, and collision frequency of metallic impurity particles gradually increase under AC, DC, and AC/DC 1:2 voltages, which is hypothesized to be due to the motion velocity of particles being related to the effective value of the voltage. The higher the effective value of the external voltage is, the faster the average motion speed of the particles between electrodes is, and the higher the number of particles colliding with electrodes is.

### 4.4. The Effect of Particle Size on the Movement of Metallic Impurity Particles

The metallic impurity particles contained in the insulation oil in the oil passage of the transformer have different sizes of between a few and a hundred microns [23]. The movement characteristics of metallic impurity particles of different sizes in insulating oil may also be different. In order to analyze the effect of the size of metallic impurity particles on their motion characteristics, five different sizes of metallic impurity particles with particle diameters of 5 μm, 20 μm, 50 μm, 70 μm, and 100 μm were used during the simulation. The electrode applied a 10 kV voltage, and the oil flow velocity was 0.01 m/s. Plate and ball electrodes were selected for the simulation.

**Figure 10:**
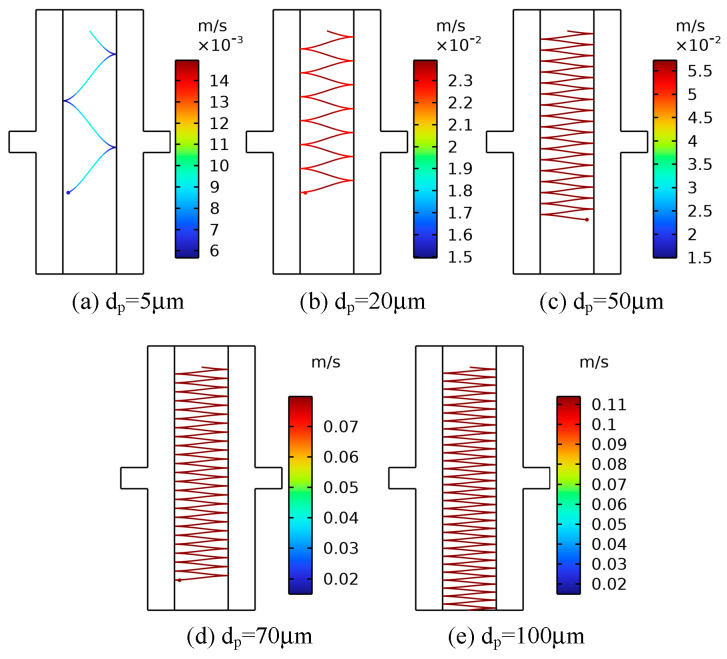
The trajectories of metallic impurity particles with different particle sizes in the plate electrode.

**Figure 11:**
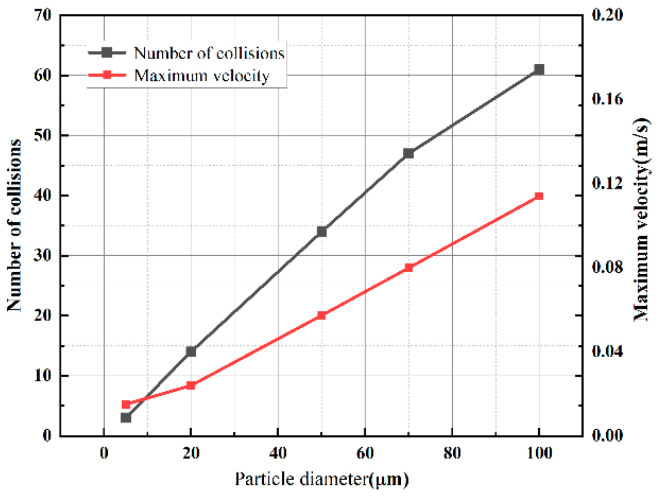
Characteristic motion parameters of metallic impurity particles with different particle sizes in plate electrode.

Figure 10 shows the trajectory diagram of metallic impurity particles of different sizes in the plate electrode. With the increase in particle size, the movement of particles between plate electrodes becomes increasingly dense. As shown in Figure 11, with the increase in the size of the metallic impurity particles, the maximum motion speed of the particles between plates increases, and the number of particles colliding with the electrode surface increases significantly. When the particle diameter is 5 μm, the maximum particle velocity is 0.015 m/s, and the number of collisions is three. When the particle diameter reaches 100 μm, the maximum particle velocity is 0.1140 m/s, which is about 7.6 times that of the 5 μm particles, and the number of collisions increases to 61, which is about 20 times that of the 5 μm particles.

**Figure 12:**
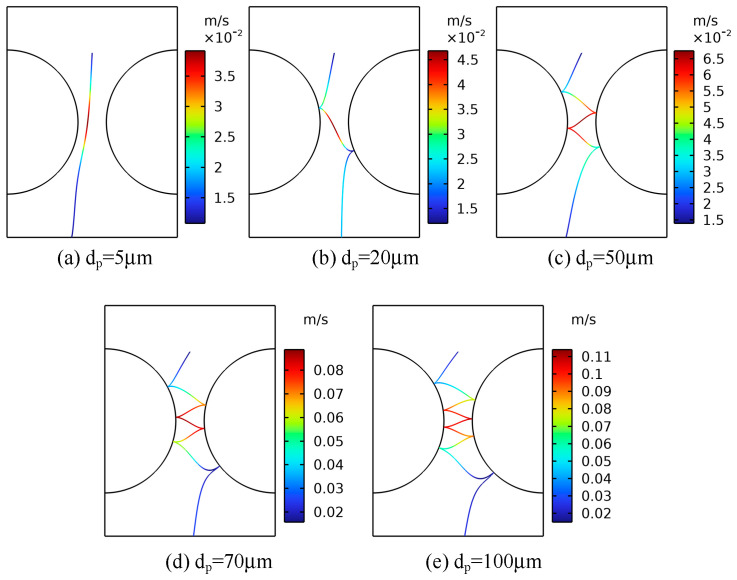
Movement trajectories of metallic impurity particles with different particle sizes in spherical electrode.

**Figure 13:**
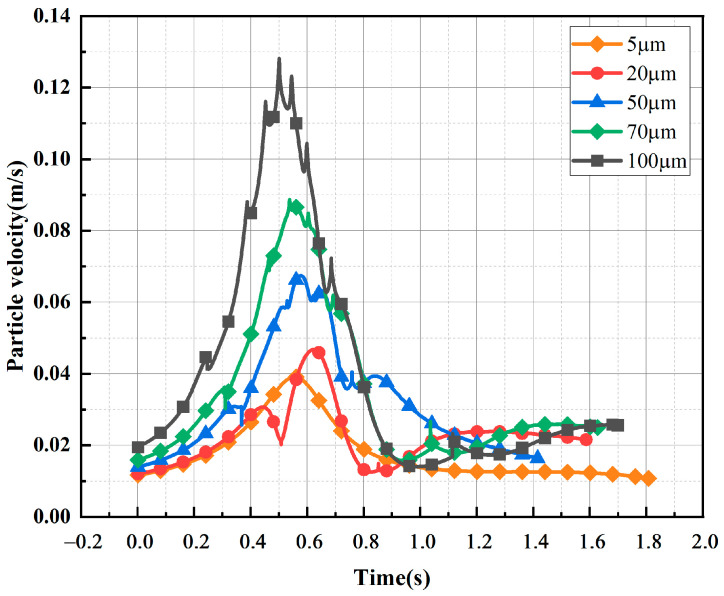
Comparison of velocities of metallic impurity particles with different particle sizes in spherical electrode.

Figure 12 shows the trajectory diagram of metallic impurity particles with different particle sizes in the ball electrode, and it can be seen that the overall motion process is similar to that of the plate electrode. With the increase in particle diameter, the number of collisions between particles and the ball electrode increases gradually. It can be seen from Figure 13 and Table 6 that under the ball electrode, the motion velocity of the metallic impurity particles first increases and then decreases, and the maximum motion velocity appears at the central axis of the ball electrode. This is because the electric field is not uniform under the ball electrode, resulting in the maximum field strength being found at the central axis of the ball electrode. As the diameter of a particle increases, its maximum velocity also increases. At 5 μm, the maximum particle velocity is 0.0362 m/s. At 20 μm, the particle’s maximum velocity is 0.0468 μm. When the particle diameter is 100 μm, the maximum velocity of the particle is as high as 0.1282 m/s, which is much higher than the maximum velocity when the particle diameter is 5 μm. At the same time, with the increase in particle diameter, the time required for a particle to move to the axial boundary first decreases and then increases. When the particle size is 5 μm, the time required to move to the axial boundary is 1.808 s; when the particle size is 50 μm, the time is reduced to 1.415 s; and when the impurity particle’s size reaches 100 μm, the time required for moving to the axial boundary is increased to 1.699 s.

According to our analysis of the simulation results, the motion velocity of solid particles under an electric field is mainly affected by the size of the external electric field and the particle size. Under conditions of low oil flow velocity, the particle motion is primarily governed by the electric field force and the viscous resistance of the oil. From the analysis of Equations (1), (2) and (5), it is evident that the electric field force is proportional to the square of the particle radius, while the viscous resistance is proportional to the particle radius. Thus, the impact of the particle radius on the electric field force is more significant than its effect on the viscous resistance. As a result, an increase in particle radius leads to a greater electric field force acting on the metallic impurity particles, which accelerates their transverse motion, increases the frequency of their round-trip movement between the plates, and raises the number of collisions with the electrode. At the same time, as the particle size increases, it can be seen from Equation (3) that the resultant force of gravity and the buoyancy force on the particle increase, so the longitudinal velocity of the particle increases, and the time that it takes for the particle to move to the axial boundary decreases. However, with the increasing size of the particle, the transverse motion speed of the particle will also be faster and faster, resulting in the viscous resistance of the particle gradually increasing, and eventually, the longitudinal velocity of the particle will slow down, so that the time that it takes for the particle to move to the axial boundary will increase.

### 4.5. The Effect of Oil Flow Velocity on the Movement of Metallic Impurity Particles

For transformers with different capacities and voltage levels, the internal oil passage structures are also different, and the types of oil pumps equipped are different. The oil flow velocity in the transformer is generally below 1 m/s, and the average oil flow velocity can reach 0.2 m/s for transformers with larger capacities and higher voltage levels [30]. The insulating oil flow rate in the transformer is essentially no higher than 0.5 m/s, but the flow speed around the oil pump may be higher [39]. To investigate the effect of the oil flow rate on the motion characteristics of metallic impurity particles, the flow rate was set as the only variable, a 10 kV voltage was applied to the electrode, and the particle diameter was 50 μm. Five oil flow speeds were selected, namely, 0.01 m/s, 0.02 m/s, 0.1 m/s, 0.2 m/s, and 0.5 m/s. Plate and ball electrodes were selected for the simulation.

**Figure 14:**
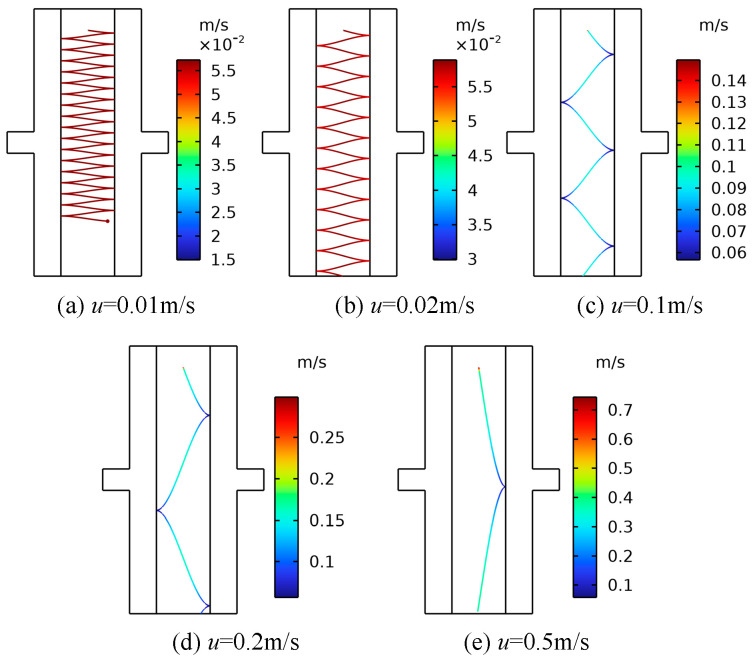
The motion trajectories of metallic impurity particles at different oil flow rates in the plate electrode.

**Figure 15:**
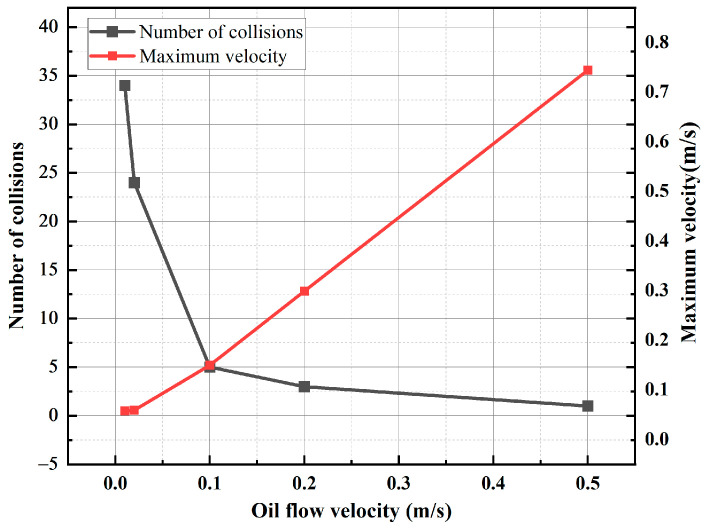
The characteristic motion parameters of metallic impurity particles at different oil flow rates in the plate electrode.

As can be observed in Figure 14 and Figure 15, an increase in the oil flow rate under the plate electrode results in a higher maximum velocity of the metallic impurity particles and a reduction in the number of collisions with the electrode. When the oil flow velocity reaches 0.5 m/s, the particles only collide with the plate once. Additionally, a higher oil flow rate causes the particles to reach the boundary of the model sooner.

Subsequently, the motion characteristics of metal particles under various oil flow rates in the spherical electrode are simulated. The results are presented in Table 7.

It can be seen from Table 7 that under the ball electrode, with the increase in the oil flow velocity, the change in the maximum velocity of the metallic impurity particles and the change in the number of collisions with the plate are consistent with the change in the plate electrode. Under the spherical electrode, when the oil flow rate reaches 0.1 m/s, the number of collisions between the metal particles and the electrode has been reduced to zero. When the oil flow velocity is 0.01 m/s, the particles move to the boundary at 1.415 s. With the increase in the oil flow velocity, the time required for the particles to move to the boundary becomes shorter. When the oil flow rate is 0.5 m/s, the time required for the particles to move to the boundary is 0.027 s.

Based on the analysis of the results, it is evident that changes in oil flow velocity do not significantly affect the transverse motion of metallic impurity particles. However, the oil flow velocity has a substantial impact on the longitudinal motion of these particles. In a high-speed oil flow, the drag force predominantly influences the metal particles. The particles, initially at rest, are rapidly accelerated by the drag force. Due to the high oil flow velocity and the resulting strong drag force, particles can move quickly through the region of high electric field gradients, leading to fewer collisions with the plate. Therefore, increasing the oil flow speed reduces the likelihood of metallic impurity particles transferring charge between electrodes and decreases the chance of agglomeration. This helps minimize the formation of small particle bridges and prevents abnormal discharge that could lead to insulation failure.

### 4.6. Effect of Dynamic Viscosity of Insulating Oil on Movement of Metallic Impurity Particles

Dynamic viscosity is a crucial parameter for characterizing the flow behavior of transformer oil, and its variation is primarily influenced by the degree of insulation oil deterioration and the temperature of the insulation oil. On the one hand, prolonged operation of the transformer can lead to the degradation of the physical and chemical properties of the insulating oil, which adversely affects its overall insulation performance. The dynamic viscosity reflects the degree of deterioration of the oil. Pure insulation oil usually has a low dynamic viscosity, and industry standards require that the dynamic viscosity of the oil at 40 °C is about 0.006 Pa·s to 0.013 Pa·s. As the oil ages, the content of impurities (such as furfural and acid) in the oil increases, resulting in a large number of polar substances that gradually increase the viscosity [32]. On the other hand, when the temperature increases, the cohesion between the insulating oil molecules will decrease, resulting in the dynamic viscosity of the insulating oil decreasing. Affected by the external environment and operating conditions, the transformer oil temperature fluctuates to a certain extent, which may affect the motion characteristics of the metallic impurity particles [40]. Therefore, this section analyzes the effects of the oil’s quality and temperature on the motion characteristics of metal particles suspended in oil, with a focus on the dynamic viscosity. In the simulation process, a metallic impurity particle with a diameter of 50 μm was set, a voltage of 10 kV was applied to the electrode, and the oil flow speed was 0.01 m/s. The motion characteristics of metallic impurity particles were analyzed at 0.006 Pa·s, 0.01 Pa·s, 0.015 Pa·s, and 0.02 Pa·s, respectively. Plate and ball electrodes were selected for the simulation.

**Figure 16:**
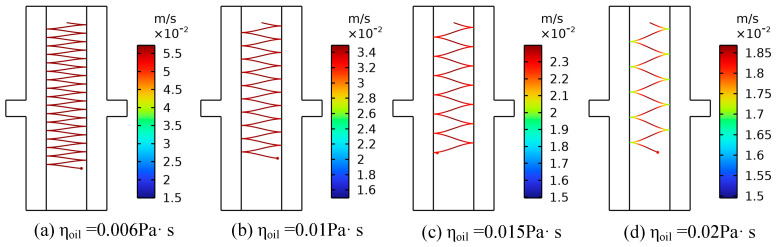
Movement trajectories of metallic impurity particles in plate electrode under different dynamic viscosities.

**Figure 17:**
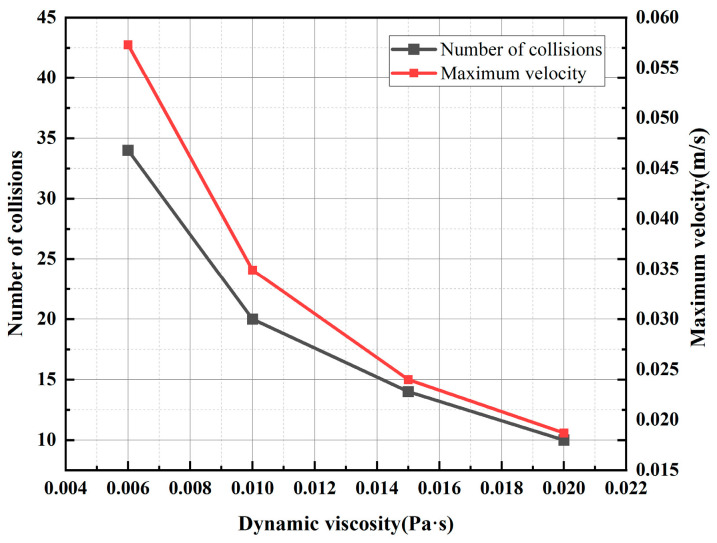
Characteristic motion parameters of metallic impurity particles in plate electrode under different dynamic viscosities.

Figure 16 and Figure 17, respectively, show the trajectory diagram of metallic impurity particles in the plate electrode and the characteristic motion parameters of metallic impurity particles under different dynamic viscosities. As the dynamic viscosity of the insulating oil increases, the trajectory of the metallic impurity particles in the plate electrode becomes more dispersed, the number of collisions between the particles and the electrode decreases significantly, and the maximum particle motion speed also declines. However, as the dynamic viscosity continues to increase, the rate of decrease in both the maximum motion velocity and the number of collisions with the electrode gradually slows down.

The motion characteristics of metal particles under varying dynamic viscosities in a spherical electrode are examined. Similar to the plate electrode, with increasing dynamic viscosity, the maximum velocity of the metal particles and the number of collisions with the electrode show comparable trends. Table 8 presents the characteristic motion parameters of metallic impurity particles in the spherical electrode at different dynamic viscosities. When the dynamic viscosity of the insulation oil is 0.02 Pa·s, the maximum particle velocity is 0.0436 m/s, and the number of collisions with the electrode is one. At this viscosity, the maximum particle velocity and number of collisions are reduced by 64.69% and 25%, respectively, compared with values observed at a lower dynamic viscosity of 0.006 Pa·s.

From the analysis of these results, it is clear that, at low oil flow rates, the particle movement is primarily influenced by the electric field force and viscous resistance. As the dynamic viscosity of the insulating oil increases, a higher viscosity leads to increased viscous resistance, which, according to Equation (5), indirectly reduces the electric field force acting on the particles and decreases their transverse motion speed. Meanwhile, Equation (4) indicates that a rising dynamic viscosity also increases the drag force on the particles. This enhanced drag force boosts the particles’ longitudinal velocity, resulting in a more dispersed trajectory within the electrode and a reduction in the number of collisions between the metallic impurity particles and the electrode.

## 5. Conclusions

The focus of this paper is an analysis of the stress of metallic impurity particles in insulation oil under an oil flow state. The COMSOL simulation analysis method is used to simulate the motion characteristics of and influencing factors on metallic impurity particles under different electric field conditions, particle sizes, oil flow speeds, and insulation oil quality conditions, and to obtain the particle motion trajectories under these different conditions. According to the simulation analysis results, this paper mainly draws the following conclusions:

(1) The motion paths of metallic impurity particles are obviously different under different electric field distributions. Under the action of an electric field, the particles will move towards the high-field-intensity region, and the motion process under the plate electrode is relatively stable and regular, while the velocity fluctuation is small. When a particle is close to the surface of the ball electrode, its motion velocity is higher, but when it is far away from the surface of the ball electrode, the motion velocity decreases. The motion velocity of particles under the pin–plate electrode changes the most, and particles will move towards the tip of the needle after colliding with the plate electrode.
(2) In an alternating current field, metallic impurity particles generally do not collide with the electrode. However, as the electric field intensity and particle size increase, the trajectories of the metallic impurity particles between the electrodes become denser, resulting in more frequent collisions between the particles and the electrodes, as well as higher motion speeds. At higher oil flow velocities, the number of collisions between metallic impurity particles and the electrode decreases, which reduces the likelihood of particle agglomeration.
(3) When the temperature of the insulation oil changes and the quality deteriorates, its dynamic viscosity will change. With the decrease in the dynamic viscosity of the insulation oil, the movement of the metallic impurity particles between the electrodes is denser, the collision times between the particles and the electrodes increases, and the maximum motion speed of the particles becomes higher.
(4) Through the analysis of the particle motion characteristics, it can be concluded that particle accumulation occurs readily in the non-uniform high-field-intensity region, and the possibility of particle accumulation is greatly reduced in the AC electric field environment. At the same time, in order to avoid the accumulation of impurity particles to form a small bridge of impurities, it is necessary to avoid significant agglomeration of impurity particles while at the same time increasing the oil flow rate appropriately and choosing a reasonable dynamic viscosity for the insulation oil. Therefore, it is necessary to monitor the oil quality and take certain filtration and purification measures to filter out large particles of impurities in time to avoid the accumulation of impurity particles to ensure the insulation performance of the oil.
(5) This paper mainly focuses on the analysis of the vertical channel model, and in transformers, the horizontal channel model is also very common. At the same time, this paper only takes a single particle as its research object in the model. In the current research, although various models for the force in the electric field are developed, it remains a problem that the understanding of the charging process of particles is not sufficiently thorough. The charging mechanism of particulate matter in oil is complex, and the influencing factors are diverse. Even in a constant electric field environment, when there are multiple particles, there are also secondary charging and charge transfer phenomena due to particle flow collisions and interactions between particles, which usually manifest as dynamic processes, and the time-variable characteristics are more obvious in an AC field or transient field. Therefore, the question of how to fully consider the dynamic characteristics of the particle flow itself and establish a time-variable charge model with more details is a topic for future research. In subsequent studies, this model will continue to be enriched, taking into account the interactions between particles, focusing on the correlation characteristics of particle clusters, and analyzing the vertical and horizontal channels separately.

To sum up, this study was able to determine the key areas for impurity detection in transformer oil, especially the area of the strongest electric field, by studying the law of motion of metallic impurity particles in different electric field environments. By monitoring the temperature and quality changes of the insulating oil, the influence of viscosity changes on the motion characteristics of impurity particles can be predicted in advance, and measures can be taken to maintain the quality of the oil and ensure the insulation performance of the transformer. Through dynamic analysis of the movement of metallic impurity particles under different conditions, the degree of pollution in transformer oil can be assessed more accurately, providing theoretical guidance for its maintenance and overhaul.

## Figures and Tables

**Table 1:** The main forces of particles and their relative magnitudes.

Type of Force	Source	Numerical Value (N)
Dragging force (*F_D_*)	Fluid motion	1.92 × 10^−5^
Magnus force (*F_M_*)	Transverse velocity gradient of particles	1.21 × 10^−6^
Saffman force (*F_S_*)	Transverse velocity gradient of particles	4.82 × 10^−8^
Gravity (*G*)	Particle property	1.08 × 10^−6^
Buoyancy (*F*)	Particle property	1.18 × 10^−7^
Electric field force (*F_e_*)	Particle charge and electric field	8.8 × 10^−6^
Pressure gradient force (*F_P_*)	Pressure gradient	1.2 × 10^−8^
Basset force (*F_B_*)	Relative acceleration between solids and flows	10^−9^–10^−7^
Additional mass inertial force (*F_Vm_*)	Relative acceleration between solids and flows	6 × 10^−9^

**Table 2:** Material properties.

Material Name	Relative Dielectric Constant	Density (g/cm^3^)	Electrical Resistivity (Ω·m)	Particle Size (μm)
Insulating oil	2.2	0.89	10^13^	—
Electrode	10^5^	8.70	1.7 × 10^−8^	—
Metallic impurity particle	10^5^	8.70	1.7 × 10^−8^	5~100

**Table 3:** Characteristic movement parameters of metallic impurity particles under different electrodes.

Electrode Type	Maximum Motion Speed (m/s)	Average Velocity (m/s)	Number of Collisions
Plate electrode	0.0573	0.0570	34
Ball electrode	0.0674	0.0320	4
Pin–plate electrode	0.2565	0.0200	5

**Table 4:** Characteristic motion parameters of metallic impurity particles in spherical electrode under different electric field intensities.

Electrode Grade (kV)	Maximum Velocity (m/s)	Average Velocity (m/s)	Number of Collisions	Time to Axial Boundary (s)
5	0.0398	0.0266	1	1.245
10	0.0674	0.0320	4	1.415
12	0.0897	0.0394	6	1.42
15	0.1443	0.0352	10	2.387

**Table 5:** Characteristic movement parameters of metallic impurity particles under different voltage types.

Plate Type	Voltage Type	Effective Voltage Value (kV)	Maximum Velocity (m/s)	Average Velocity (m/s)	Number of Collisions
Plate electrode	Direct current	10	0.0573	0.0570	11
	Alternating current	10	0.0805	0.0520	0
	AC and DC 1:2	22.36	0.4325	0.2533	51
Ball electrode	Direct current	10	0.0674	0.0320	4
	Alternating current	10	0.0882	0.0413	0
	AC and DC 1:2	22.36	0.4318	0.1659	23

**Table 6:** Characteristic motion parameters of metallic impurity particles with different particle sizes in spherical electrode.

Particle Diameter (μm)	Maximum Motion Speed (m/s)	Average Speed (m/s)	Number of Collisions	Time to Axial Boundary (s)
5	0.0392	0.0178	0	1.808
20	0.0468	0.0227	2	1.587
50	0.0674	0.0320	4	1.415
70	0.0887	0.0347	6	1.628
100	0.1282	0.0404	8	1.699

**Table 7:** Characteristic motion parameters of metallic impurity particles at different oil flow rates in spherical electrodes.

Oil Flow Velocity (m/s)	Maximum Motion Speed (m/s)	Average Speed (m/s)	Number of Collisions	Time to Axial Boundary (s)
0.01	0.0674	0.0320	4	1.415
0.02	0.0936	0.0568	2	0.636
0.1	0.3408	0.2146	0	0.149
0.2	0.6566	0.4821	0	0.066
0.5	1.5862	1.1790	0	0.027

**Table 8:** Characteristic motion parameters of metallic impurity particles in spherical electrode under different dynamic viscosities.

Dynamic Viscosity of Insulating Oil (Pa·s)	Maximum Motion Speed (m/s)	Average Speed (m/s)	Number of Collisions
0.006	0.0674	0.0320	4
0.01	0.0518	0.0163	3
0.015	0.0486	0.0203	2
0.02	0.0436	0.0196	1

## Data Availability

Data will be made available upon request.
